# Adverse childhood experiences and comorbidity in a cohort of people who have injected drugs

**DOI:** 10.1186/s12889-022-13369-5

**Published:** 2022-05-16

**Authors:** David W. Sosnowski, Kenneth A. Feder, Jacquie Astemborski, Becky L. Genberg, Elizabeth J. Letourneau, Rashelle J. Musci, Ramin Mojtabai, Lisa McCall, Eileen Hollander, Lynnet Loving, Brion S. Maher, Gregory D. Kirk, Shruti H. Mehta, Jing Sun

**Affiliations:** 1 Department of Mental Health, Bloomberg School of Public Health, Johns Hopkins University, Baltimore, MD USA; 2 Department of Epidemiology, Bloomberg School of Public Health, Johns Hopkins University, Baltimore, MD USA; 3 Johns Hopkins University School of Medicine, Baltimore, MD USA

**Keywords:** Adverse childhood experiences, Comorbidity, Substance use

## Abstract

**Background:**

Childhood adversity is associated with the onset of harmful adult substance use and related health problems, but most research on adversity has been conducted in general population samples. This study describes the prevalence of adverse childhood experiences in a cohort of people who have injected drugs and examines the association of these adverse experiences with medical comorbidities in adulthood.

**Methods:**

Six hundred fifty three adults were recruited from a 30-year cohort study on the health of people who have injected drugs living in and around Baltimore, Maryland (Median age = 47.5, Interquartile Range = 42.3–52.3 years; 67.3% male, 81.1% Black). Adverse childhood experiences were assessed retrospectively in 2018 via self-report interview. Lifetime medical comorbidities were ascertained via self-report of a provider diagnosis. Multinomial logistic regression with generalized estimating equations was used to examine the association between adversity and comorbid conditions, controlling for potential confounders.

**Results:**

Two hundred twelve participants (32.9%) reported 0–1 adverse childhood experiences, 215 (33.3%) reported 2–4, 145 (22.5%) reported 5–9, and 72 (11.1%) reported ≥10. Neighborhood violence was the most commonly reported adversity (48.5%). Individuals with ≥10 adverse childhood experiences had higher odds for reporting ≥3 comorbidities (Adjusted Odds Ratio = 2.9, 95% CI = 1.2 – 6.8, *p* = .01).

**Conclusions:**

Among people who have injected drugs, adverse childhood experiences were common and associated with increased occurrence of self-reported medical comorbidities. Findings highlight the persistent importance of adversity for physical health even in a population where all members have used drugs and there is a high burden of comorbidity.

**Supplementary Information:**

The online version contains supplementary material available at 10.1186/s12889-022-13369-5.

## Background

Over the past three decades, adverse childhood experiences – including exposure to maltreatment, household dysfunction, or other forms of trauma in childhood – have received growing attention from public health professionals as major contributors to the burden of chronic disease [1]. Seminal research in 1998 established that adverse childhood experiences are a risk factor for a wide range of mental, behavioral, and physical health problems, including many of the leading causes of death [2]. Subsequent research found that the type of health problems most strongly and consistently linked to a history of childhood adversity are substance use problems and substance use disorder, specifically opioid use disorder [3–6]. This association between childhood adversity and harmful substance use is particularly relevant in the United States, where there is an ongoing epidemic of drug-related problems, driven largely by the misuse of opioids [7].

Although adverse childhood experiences are a well-established risk factor for the onset of adult opioid use, other substance use disorders, and risky substance use behaviors such as injecting drugs, most research on childhood adversity has been conducted in insured or general population cohorts in which people with substance use disorders may be underrepresented [3]. By contrast, to our knowledge, no cohort studies have examined how a history of adverse childhood experiences affects the health of people who have injected drugs, specifically, over the course of their lives, even though adverse childhood experiences are a well-known risk factor for initiation of harmful drug use.

Of particular importance is understanding the association of childhood adversity with chronic physical illness among people who have injected drugs. Previous studies have demonstrated that mortality and morbidity are higher among people who have injected drugs than in the general population [8, 9], and that chronic-disease related deaths are a substantive and increasing contributor to overall mortality among people who use drugs, particularly as HIV-related causes of death have declined [10]. However, although there is evidence from the general population that cumulative adverse childhood experiences are associated with increased odds for physical health comorbidities (e.g., diabetes, coronary heart disease) [11, 12], it remains unclear whether adverse childhood experiences independently confer higher risk for physical health comorbidities among people who use drugs. This is because the strongest and most consistent adult corollary of childhood adversity is harmful substance use [3]; To the extent that risk for physical illness following childhood adversity may be mediated by initiating harmful substance use, it is unclear whether this association would persist in a population where all members are already known to have initiated illicit drug use. Lastly, it is important to adjust for other possible confounders and/or mediators in the association between childhood adversity and adult chronic disease. For example, sociodemographic factors like socioeconomic status, and clinical characteristics such as HIV infection have been implicated in associations with both adverse childhood experiences and adult chronic disease [13, 14]. Adjusting for these additional factors can provide a clearer picture of the independent association between adverse childhood experiences and comorbid conditions.

There were two objectives for the present study. The first objective was to describe the prevalence of self-reported adverse childhood experiences within a sample of adults in Baltimore, Maryland who have injected drugs, and to compare them to the prevalence of adverse childhood experiences in the community (i.e., Baltimore City and the state of Maryland). The second objective was to examine the association between adverse childhood experiences and chronic physical health comorbidities among this same group of individuals.

## Methods

### Study sample

Study participants were a subset of individuals from the AIDS Linked to the Intravenous Experience (ALIVE) study. Complete details of the ALIVE study, which began recruitment in 1988, are described elsewhere [15]. Briefly, the ALIVE study is an ongoing, community-based, prospective cohort study of people who have a lifetime history of injection drug use, living in and around Baltimore, Maryland. Approximately 2,600 adults were originally recruited in 1988, and subsequent waves of recruitment occurred in 1994–1995, 2005–2008, and 2015–2018. Participants attend twice-annual study visits where they complete a physical exam; complete a standardized interviewer-administered and audio-computer assisted surveys about drug use, related health-risk behaviors, and physical and mental health outcomes; and provide a blood sample for HIV, Hepatitis C (HCV) antibody testing, and other biomarker measurements.

Detailed methods for the adverse childhood experiences sub-study, data collection, and survey instruments can be found in Additional file 1: Appendix A. All ALIVE participants were eligible to participate in the adverse childhood experiences sub-study if they attended a study visit during the period of August 1^st^ 2018 – December 31^st^ 2019 and had attended at least one prior ALIVE study visit. Of the 1,127 eligible participants, 735 were recruited for this non-selective convenience sample. A total of 653 participants from three separate recruitment cohorts provided complete data on adverse childhood experiences and were included in the present analysis (see Additional file 1: Appendix B). We compared the analytic sample (*n* = 653) to those individuals missing any adverse childhood experience data (*n* = 69) on demographic characteristics (i.e., age, sex, race [Black vs. white], past six-month homelessness and income) and other study characteristics (past six-month injection drug use, HCV and HIV status, cohort group). Compared to those with complete data on adverse childhood experiences, there was a higher percentage of participants who were HIV positive among participants with missing data on childhood adversity, χ^2^ (1) = 5.1, *p* = 0.02. There were no other differences between participants with and without complete adversity data (see Additional file 1: Appendix C). The Johns Hopkins University Institutional Review Board has continuously approved the ALIVE study and approved the protocol for this sub-study. All study protocol was conducted in accordance with Johns Hopkins University Institutional Review Board’s standards and the Declaration of Helsinki. All participants provide written informed consent upon study entry and provided additional consent to participate in this sub-study.

### Measures

ALIVE cohort participants are asked at each study visit “Has a doctor or other health care provider ever said you have [condition name].” All conditions assessed – which included diabetes, hypertension, or cerebrovascular, cardiovascular, renal, chronic lung, cancer, or liver disease – were included in this analysis. One additional comorbidity – obesity (defined as a body mass index ≥ 30) – was also calculated and included in this analysis based on measurements taken during the study visit physical exam. These conditions were selected based on known associations with adverse childhood experiences (e.g., obesity) [16], prior comorbidity indices created in the ALIVE cohort [17], and medical conditions included in comorbidity burden indices (e.g., Charlson Comorbidity Index) [18]. Based on the distribution of comorbidities in the study sample, scores were categorized into the following groups: 0 comorbidities, 1–2 comorbidities, and ≥ 3 comorbidities (see Table 1). Within ALIVE, this measure of comorbidity burden has been independently associated with geriatric phenotypes, frailty transitions, hospitalization, and mortality [17, 19, 20].

**Table 1:** Baseline Characteristics of ALIVE Participants by their ACE Burden

		ACE Category			
Variable	Overall	0–1 ACE (32.9%)	2–4 ACEs (33.3%)	5–9 ACEs (22.5%)	≥10 ACEs (11.1%)
Age: Median (IQR)	48 (42–52)	49 (43–53)	49 (43–53)	46 (41–51)	46 (40–52)
Sex: N (% male)	440 (67)	147 (69)	158 (72)	98 (67)	37 (51)
Race: N (% Black)	530 (81)	185 (86)	180 (82)	109 (74)	56 (78)
HIV status (% positive)	140 (21)	47 (21)	43 (19)	33 (22)	17 (23)
HCV status: N (% positive)	501 (77)	151 (71)	182 (83)	112 (76)	56 (78)
Current IDU: N (%)	401 (61)	127 (59)	128 (58)	95 (65)	51 (71)
Homelessness: N (%)	164 (25)	40 (19)	51 (23)	46 (31)	27 (38)
Income < $5,000: N (%)	504 (79)	164 (78)	167 (78)	112 (76)	61 (86)
Cohort 1: N (%)	252 (39)	88 (41)	92 (42)	51 (35)	21 (29)
Cohort 2: N (%)	173 (26)	58 (27)	52 (24)	44 (30)	19 (26)
Cohort 3: N (%)	228 (35)	68 (32)	76 (35)	52 (35)	32 (44)
0 Comorbidities: N (%)	160 (25)	51 (31)	56 (35)	36 (22)	17 (10)
1–2 Comorbidities: N (%)	358 (55)	123 (34)	116 (32)	82 (22)	37 (10)
≥3 Comorbidities: N (%)	126 (20)	38 (30)	43 (34)	27 (21)	18 (14)

Race was coded as Black versus white. Cohort 1 = recruitment period before 2005; Cohort 2 = recruitment period 2005–2014; Cohort 3 = recruitment period 2015–2018. Values by ACE category reflect conditional probabilities

*Note*. *N* = 653, *IDU* Injection drug use, *HCV* Hepatitis C serologic status, *ACE* Adverse childhood experience

Participants’ childhood exposure to adversity was assessed using a modified version of the adverse childhood experiences questionnaire (Additional file 1: Appendix D) [21]. Briefly, the questionnaire included 21 questions assessing 14 adverse childhood experiences occurring before the age of 18 years: physical and emotional neglect, physical, emotional, and sexual abuse, loss of a parent, domestic violence, parent substance abuse, parent mental illness, incarceration of a household member, bullying, social ostracization, neighborhood violence, and poverty. For adversities assessed with multiple items, endorsing any one of those items was sufficient to indicate the presence of that adversity. Scores were summed and then categorized into the following groups: 0–1 adversities, 2–4 adversities, 5–9 adversities, ≥ 10 adversities. These cut-offs were based on the distribution of ACEs in the current sample. Specifically, “0–1 adversities” was chosen as the reference category given the large percentage of the sample that reported exposure to at least one adverse childhood experience (~ 50%); other categories were selected based on what we deemed to be adequate group sizes that also reflected conceptually different adversity exposure groups.

We also adjusted for several sociodemographic and clinical characteristics that may confound (e.g., age, sex) or mediate (e.g., HIV status, injection drug use) the association between childhood adversity and adult chronic disease. Sociodemographic characteristics included self-reported age, race (Black versus White), sex, cohort group, past six-month income below $5,000 and past six-month homelessness. Clinical characteristics included self-reported HIV status, anti-HCV status (from antibody test), and past six-month injection drug use.

### Statistical analysis

First, we described the prevalence of adverse childhood experiences in the ALIVE cohort and compared these data with data from Maryland adults (state-wide and in Baltimore City) who participated in the 2018 Maryland Behavioral Risk Factors Surveillance System (BRFSS), which contained an adverse childhood experiences module [22]. We also examined the distribution and co-occurrence (i.e., conditional probabilities) of adverse childhood experiences in our sample. We then evaluated the association between adverse childhood experiences and comorbid conditions. During exploratory analysis, we tested the crude association between adverse childhood experiences and medical comorbidities using an ordinal logistic regression model with robust standard errors. Since the data violated the proportional odds assumption (χ^2^(3) = 40.23, *p* < 0.001), multinomial logistic regression with robust standard errors was selected for final models. After a crude model (Model A) was tested without adjustment for covariates, a second model (Model B) was tested, adjusting for demographic characteristics (i.e., age, race [Black vs. White], sex, cohort) and socioeconomic indicators (i.e., income below $5,000/year, past six-month homelessness). The third model (Model C) additionally controlled for current anti-HCV status and HIV status (yes/no), and past six-month injection drug use (yes/no). Analyses were organized in this way to attempt to tease apart whether sociodemographic or clinical characteristics were influencing the association between childhood adversity and adult comorbid conditions. Models will be referred to herein as Model A, B, and C. Covariates in the adjusted model were selected based on known associations with adverse childhood experiences and the crude association with the outcome. Lastly, a sensitivity analysis was conducted with all recruited participants (*n* = 722), with missing responses for adverse childhood experience items recoded to 0 (i.e., no exposure). This approach was used since minimal adversity data were missing (i.e., < 10% of the sample), and when assuming a response of “yes” to the missing adversity items, few participants (*n* = 26) moved from on adversity category to another based on our categorical coding strategy. All statistical tests used a *p*-value threshold of 0.05. All analyses were conducted using Stata version 15 [23].

## Results

The analytic sample included 653 individuals (Median age = 47.5, Interquartile Range [IQR] = 42.3–52.3 years; 67.3% male, 81.1% Black) with a history of injection drug use. Participants had a median of 11.6 person-years of follow-up (IQR = 3.0–13.8, range = 0–14.6). Table 1 presents the baseline characteristics and comorbidity burden of participants by the number of adverse childhood experiences. Most participants (55%) reported 1–2 comorbidities; 25% of participants reported zero medical comorbidities and 20% reported at least 3 medical comorbidities. The most common comorbidity pattern in the sample is having no conditions (33%). Among participants with exactly one condition, the most common condition is hypertension (15% of the full sample). Among participants with exactly two comorbidities, the most common unique condition combination is hypertension and obesity (6%). Among participants with three or more comorbidities, the most common unique combination is hypertension, obesity, and diabetes (3%). A detailed breakdown of the most common, unique comorbidities in the sample can be found in Additional file 1: Appendix F.

Participants reported a median of 3 adverse childhood experiences (IQR = 1–6, range = 0–14); 212 Participants (32.9%) reported 0–1 adverse childhood experience, 215 (33.3%) reported at 2–4, 145 (22.5%) reported 5–9, and 72 (11.1%) 10 or more. The most reported adverse childhood experience was growing up in a violent neighborhood (48.5%), followed by living in poverty (39.5%), having a household member with a substance use disorder (36.9%), and loss of a parent [due to divorce, abandonment, or some other reason] (34.7%). For every source of adversity assessed that had a comparable measure in Maryland’s 2018 BRFSS survey except one (emotional abuse), self-reported adversity was more common in the ALIVE cohort than among Maryland adults in Baltimore City and all adults state-wide (Table 2). The most commonly co-occurring adverse childhood experiences were poverty and neighborhood violence, with 29.4% of participants who reported living in poverty also reported growing up in a violent neighborhood (Table 3). Other commonly co-occurring adverse childhood experiences were neighborhood violence and household member substance use disorder (24.5%), neighborhood violence and loss of a parent (22.3%), social ostracization and emotional neglect (21.7%), and poverty and household member substance use disorder (21.4%).

**Table 2:** Prevalence of Adverse Childhood Experiences in ALIVE and Maryland Adults

Adversity	ALIVE	Baltimore City	Maryland
Emotional Neglect	26.8%	N/A	N/A
Physical Neglect	13.3%	N/A	N/A
Loss of a Parent	34.7%	N/A	N/A
Domestic Violence in the Home	23.7%	20.5%	15.3%
Household Member Substance Use	36.9%	36.6%	24.8%
Household Member Mental Illness	22.9%	22.3%	15.4%
Household Member Incarcerated	26.4%	17.3%	8.0%
Bullying	20.5%	N/A	N/A
Social Ostracization	28.1%	N/A	N/A
Neighborhood Violence	48.5%	N/A	N/A
Poverty	39.5%	N/A	N/A
Emotional Abuse	21.2%	34.6%	34.0%
Physical Abuse	20.5%	15.5%	14.7%
Sexual Abuse	19.6%	14.4%	12.0%
Parent Divorce	N/A	45.8%	29.1%

*Note*. *N* = 653. "Loss of Parent" item in ALIVE questionnaire includes "divorce" as part of the question. Maryland estimates are based on 2018 Maryland Behavioral Risk Factor Surveillance System (BRFSS) ACE Module

**Table 3:** Co-Occurrence of Adverse Childhood Experiences in ALIVE

Adversity	1	2	3	4	5	6	7	8	9	10	11	12	13
1. Emotional Neglect	-												
2. Physical Neglect	10.5	-											
3. Loss of Parent	15.3	9.1	-										
4. Domestic Violence	13.6	8.1	14.5	-									
5. Parent Substance Use	16.6	11.0	19.1	17.7	-								
6. Parent Mental Illness	15.0	8.2	13.0	12.2	16.2	-							
7. Parent Incarcerated	9.4	5.9	12.5	8.8	14.4	8.5	-						
8. Bullying	13.6	6.2	11.7	9.1	12.5	10.2	8.7	-					
9. Social Ostracization	**21.7**	11.0	15.7	12.7	17.4	15.3	9.8	14.0	-				
10. Neighborhood Violence	16.5	9.9	**22.3**	16.2	**24.5**	14.8	19.9	13.1	17.9	-			
11. Poverty	16.3	10.7	21.4	16.3	**21.4**	14.7	15.6	13.1	17.7	**29.4**	-		
12. Emotional Abuse	15.0	8.8	12.5	12.4	15.7	13.0	7.5	10.2	15.3	14.7	13.0	-	
13. Physical Abuse	13.9	7.9	11.1	11.3	14.5	11.4	6.7	9.4	14.2	14.0	12.7	16.5	-
14. Sexual Abuse	11.0	6.5	11.0	10.4	13.0	11.0	8.2	7.2	12.1	12.7	12.5	11.1	11.3

*Note. N* = 653. Values in cells are percentages reflecting conditional probabilities. **Boldface** indicates percentage over 20%

Results for each of the models (i.e., A, B, and C) are presented in Table 4. Results from Model A, which tested the crude association between adverse childhood experiences and comorbidity, demonstrated that individuals who reported experiencing ≥ 10 adverse childhood experiences had higher odds of reporting at least three comorbid conditions (Odds Ratio [OR] = 2.7; 95% CI = 1.3 – 6.0; *p* = 0.01). Similarly, results from Model B, which adjusted for sociodemographic characteristics, revealed that individuals who reported experiencing ≥ 10 adverse childhood experiences had higher odds of reporting at least three comorbid conditions (Adjusted OR = 2.7; 95% CI = 1.2 – 6.3; *p* = 0.01). This effect persisted in Model C, which additionally adjusted for HCV and HIV status and past six-month injection drug use, where individuals who reported experiencing ≥ 10 adverse childhood experiences had higher odds of reporting at least three comorbid conditions (Adjusted OR = 2.9; 95% CI = 1.2 – 6.8; *p* = 0.01). No other associations between adverse childhood experiences and comorbidity burden were statistically significant. Lastly, results of sensitivity analysis, where individuals with missing adversity data were included by coding their missing responses to 0 (i.e., no exposure; *n* = 69), were qualitatively similar to the main analysis (Additional file 1: Appendix F).

**Table 4:** Comorbidity Burden and Adverse Childhood Experiences in ALIVE (*N* = 653)

Outcomes	Exposure	Model A		Model B^a^		Model C^b^	
Comorbidity Groups	ACEs	OR (95% CI)	*p*-value	OR (95% CI)	*p*-value	OR (95% CI)	*p*-value
0 comorbid conditions (ref.)	-	-	-	-	-	-	-
1–2 comorbid conditions	0 to 1 ACEs	ref	-	ref	-	-	-
	2 to 4 ACEs	0.8 (0.6 – 1.2)	.48	0.9 (0.6 – 1.3)	.73	0.9 (0.6 – 1.3)	.75
	5 to 9 ACEs	0.9 (0.6 – 1.4)	.90	1.1 (0.7 – 1.6)	.55	1.1 (0.7 – 1.6)	.52
	≥ 10 ACEs	1.1 (0.6 – 1.9)	.68	1.0 (0.6 – 1.9)	.75	1.1 (0.6 – 1.9)	.70
≥ 3 comorbid conditions	0 to 1 ACEs	ref	-	ref	-	ref	-
	2 to 4 ACEs	1.0 (0.6 – 1.8)	.74	1.2 (0.7 – 2.1)	.45	1.2 (0.7 – 2.2)	.42
	5 to 9 ACEs	1.2 (0.7 – 2.3)	.40	1.7 (0.9 – 3.3)	.09	1.8 (0.9 – 3.4)	.07
	≥ 10 ACEs	2.7 (1.3 – 6.0)	.01	2.7 (1.2 – 6.3)	.01	2.9 (1.2 – 6.8)	.01

*Note*. *ACEs* Adverse childhood experiences, *OR* Odds Ratio, *CI* Confidence Interval

^a ^Estimates adjusted for age, race, sex, cohort, and past six-month income and homelessness

^b ^Estimates additionally adjusted for HIV and anti-HCV status, and past six-month injection drug use. Estimates are based on the use of robust standard errors

## Discussion

Using data from a long-standing, well-characterized cohort of people who have injected drugs, we demonstrate that adverse childhood experiences are associated with high burden of chronic physical health comorbidities. These findings are consistent with previous literature that reporting more adverse childhood experiences is associated with higher odds of risk behaviors and comorbid conditions including obesity, diabetes mellitus, heart diseases, and disability [11, 12]. Importantly, our findings show that this association persists even in a sample where everyone is known to have used drugs and have a high burden of chronic comorbidities [17]. Given the high risk for comorbidity among drug-using populations, it is not surprising that further risk for comorbidities is only realized at high levels of exposure to adversity (i.e., > 10 adverse childhood experiences). Nonetheless, nearly 10% of our sample endorsed at least 10 adverse childhood experiences. This suggests that exposure to childhood adversity further drives the risk of high comorbidity burden in this population. Moreover, the association we observed between high adverse childhood experiences and high physical comorbidity persists even after controlling for HIV and HCV status, and injection drug use. This suggests that other factors, in addition to these infectious and behavioral risks, mediate the association of childhood adversity with comorbid chronic disease in people who have injected drugs. Future research should explore mechanisms through which childhood adversity contributes to the burden of comorbidity.

As expected, adverse childhood experiences were common among people have injected drugs in Baltimore city. This is consistent with prior literature on the association between adverse childhood experiences and harmful substance use [3]. Even after excluding those adverse childhood experiences not assessed in the BRFSS, approximately seven out of 10 ALIVE participants reported experiencing at least one adverse childhood experience, demonstrating a high burden of adversity among persons who have injected drugs. Notably, neighborhood violence and poverty were cited as the most prevalent adverse childhood experiences, as well as the adverse childhood experiences that were most often co-occurring in the present sample. Although not included in the BRFSS adverse childhood experiences module, research evidence demonstrates that various indicators of poverty (e.g., parent income and education status, monetary resources) and neighborhood violence should be included in adverse childhood experience assessments [24, 25]. Given the high rates of endorsement for these two experiences in our sample, exclusion of these experiences may underestimate risk among similar populations with high health-risk behaviors.

Our data also replicate an important pattern seen in recent research investigations: certain adverse childhood experiences are more likely to co-occur than others [26–28]. Specifically, we found that neighborhood violence and poverty often co-occurred with experiences of losing a parent and living with someone who has a substance use disorder. These data highlight the importance of understanding the pattern of adversity experienced within and across populations. An important next step in this area would be to explore pathways linking adversity – both individual events and constellations of experiences – to harmful substance use (e.g., injection drug use). This work will clarify if the patterns of adversity noted in our sample place individuals at unique risk for injection drug use, harmful substance use more broadly, or a general high-risk phenotype. Similarly, it will be important to identify patterns of adversity exposure among other drug-using populations, and whether certain constellations of adverse childhood experiences are associated with specific drug use patterns (e.g., opioid use, polysubstance use).

### Limitations

Results from this study should be considered in the context of several limitations. First, the sample is comprised of a mostly male, mostly Black, urban, east-coast cohort, many of whom came of age during the peak of the HIV epidemic. Thus, this could limit the generalizability of the current findings. ALIVE participants are also unique in that they were recruited because they had a history of injecting drugs. Injection drug use may be a marker for severity of drug-related problems; different effects of adversity might be observed in the broader cohort of adults who use heroin or cocaine but have never injected. Third, adverse childhood experiences were assessed retrospectively. Although there is a temporal sequence from childhood adversity to chronic conditions developed later in life, recall bias might differentially impact different age groups and differentially impact associations between adverse childhood experiences and comorbidity burden. We also did not adjust for all possible confounders in the association between childhood adversity and comorbid conditions. Other social determinants of health, such as food insecurity, diet, housing, and transportation insecurity are associated with exposure to adversity and health and may influence this association. Finally, the Maryland state law requirement that we report items indicating a history of child abuse to the Baltimore Department of Social Services – and the need to notify participants of that requirement – may have led to an undercount of the number of participants who experienced childhood abuse (see Additional file 1: Appendix A). However, the number of participants who declined to respond to the childhood abuse questions was limited (*n* = 13), and characteristics of participants with and without the missing data on childhood abuse questionnaire were similar.

## Conclusions

To our knowledge, this is the first study to assess adverse childhood experiences and its association with comorbidity burden in a population of people who have injected drugs. The burden of adverse childhood experiences was high, with neighborhood violence, living in poverty, and having a parent who abused substances as common experiences for these individuals. Results highlight the importance of examining the constellation of adverse childhood experiences (in addition to individual experiences) in populations with high-risk behaviors. Moreover, at high levels of exposure to adverse childhood experiences, there was increased odds for numerous comorbid conditions; this is notable because here this association occurred even in a population where all adults have injected drugs, so initiation of drug use likely does not fully mediate this association. Future research should explore how adverse childhood experiences influence high-risk behaviors (i.e., drug use trajectories), risk for individual comorbid conditions, and the underlying mechanisms linking adverse childhood experiences with chronic diseases to identify modifiable factors that promote resilience within this population.

## Supplementary Information

**Additional file 1.**

## Data Availability

The datasets generated and/or analyzed during the current study are not publicly available due to reasons of sensitivity but are available from the corresponding author on reasonable request.

## Abbreviations

ALIVE: AIDS Linked to the Intravenous Experience
BRFSS: Behavioral Risk Factors Surveillance System
HCV: Hepatitis C serologic status
IQR: Interquartile Range
OR: Odds Ratio

## References

[1] Centers for Disease Control and Prevention. Adverse Childhood Experiences. Published 2021. Accessed 3 Aug 2021. https://www.cdc.gov/violenceprevention/aces/about.html?CDC_AA_refVal=https%3A%2F%2Fwww.cdc.gov%2Fviolenceprevention%2Facestudy%2Fabout.html

[2] Felitti VJ, Anda RF, Nordenberg D (1998). Relationship of childhood abuse and household dysfunction to many of the leading causes of death in adults: the adverse childhood experiences (ACE) study. Am J Prev Med.

[3] Hughes K, Bellis MA, Hardcastle KA (2017). The effect of multiple adverse childhood experiences on health: a systematic review and meta-analysis. Lancet Public Heal.

[4] Conroy E, Degenhardt L, Mattick RP, Nelson EC (2009). Child maltreatment as a risk factor for opioid dependence: comparison of family characteristics and type and severity of child maltreatment with a matched control group. Child Abus Negl.

[5] Naqavi MR, Mohammadi M, Salari V, Nakhaee N. The Relationship between childhood maltreatment and opiate dependency in adolescence and middle age. Addict Heal. 3(3–4):92–98. Accessed 14, July 2021. http://www.ncbi.nlm.nih.gov/pubmed/24494122%0A. http://www.pubmedcentral.nih.gov/articlerender.fcgi?artid=PMC3905531 PMC3905531 24494122

[6] Dube SR, Felitti VJ, Dong M, Chapman DP, Giles WH, Anda RF (2003). Childhood abuse, neglect, and household dysfunction and the risk of illicit drug use: The adverse childhood experiences study. Pediatrics.

[7] Opioid Overdose Crisis | National Institute on Drug Abuse (NIDA). Accessed 21 Sept 2021. https://www.drugabuse.gov/drug-topics/opioids/opioid-overdose-crisis

[8] Mathers BM, Degenhardt L, Bucello C, Lemon J, Wiessing L, Hickman M (2013). Mortality among people who inject drugs: a systematic review and meta-analysis. Bull World Health Organ.

[9] Nambiar D, Weir A, Aspinall EJ (2015). Mortality and cause of death in a cohort of people who had ever injected drugs in Glasgow: 1982–2012. Drug Alcohol Depend.

[10] Sun J, Mehta SH, Astemborski J, et al. Mortality among persons who inject drugs: a prospective cohort followed over three decades in Baltimore, Maryland, USA. Addiction. Published online August 2, 2021:add.15659. doi:10.1111/ADD.15659

[11] Anda RF, Felitti VJ, Bremner JD (2006). The enduring effects of abuse and related adverse experiences in childhood: a convergence of evidence from neurobiology and epidemiology. Eur Arch Psychiatry Clin Neurosci.

[12] Campbell JA, Walker RJ, Egede LE (2016). Associations between adverse childhood experiences, high-risk behaviors, and morbidity in adulthood. Am J Prev Med.

[13] Bellis MA, Lowey H, Leckenby N, Hughes K, Harrison D (2014). Adverse childhood experiences: retrospective study to determine their impact on adult health behaviours and health outcomes in a UK population. J Public Heal (United Kingdom).

[14] Yang HY, Beymer MR, Suen SC. Chronic disease onset among people living with HIV and AIDS in a large private insurance claims dataset. Sci Rep. 2019;9(1). doi:10.1038/s41598-019-54969-3 10.1038/s41598-019-54969-3 PMC6897968 31811207

[15] Vlahov D, Anthony JC, Muñoz A (1991). The Alive Study: a longitudinal study of HIV-1 infection in intravenous drug users: description of methods. J Drug Issues.

[16] Wiss DA, Brewerton TD (2019). Adverse childhood experiences and adult obesity: a systematic review of plausible mechanisms and meta-analysis of cross-sectional studies. Physiol Behav.

[17] Piggott DA, Bandeen-Roche K, Mehta SH (2020). Frailty transitions, inflammation, and mortality among persons aging with HIV infection and injection drug use. AIDS.

[18] Charlson ME, Pompei P, Ales KL, MacKenzie CR (1987). A new method of classifying prognostic comorbidity in longitudinal studies: development and validation. J Chronic Dis.

[19] Piggott DA, Muzaale AD, Varadhan R (2017). Frailty and cause-specific hospitalization among persons aging with HIV infection and injection drug use. Journals Gerontol - Ser A Biol Sci Med Sci.

[20] Piggott DA, Muzaale AD, Mehta SH, et al. Frailty, HIV infection, and mortality in an aging cohort of injection drug users. PLoS One. 2013;8(1). doi:10.1371/journal.pone.0054910 10.1371/journal.pone.0054910 PMC3561408 23382997

[21] Finkelhor D, Shattuck A, Turner H, Hamby S (2015). A revised inventory of adverse childhood experiences. Child Abus Negl.

[22] Maryland Behavioral Risk Factor Surveillance System. Adverse Childhood Experiences (ACEs) in Maryland: Data from the 2018 Maryland BRFSS.; 2020. Accessed 27 July 2021. http://phpa.dhmh.maryland.gov/ccdpc/Reports/Pages/brfss.aspx

[23] Stata Statistical Software: Release 17. Published online 2021.

[24] Finkelhor D, Shattuck A, Turner H, Hamby S (2015). A revised inventory of adverse childhood experiences. Child Abus Negl.

[25] Wade R, Shea JA, Rubin D, Wood J (2014). Adverse childhood experiences of low-income urban youth. Pediatrics.

[26] Woerner J, Overstreet C, Amstadter AB, Sartor CE (2020). Profiles of psychosocial adversity and their associations with health risk behaviors and mental health outcomes in young adults. J Health Psychol.

[27] Barboza GE (2018). Latent classes and cumulative impacts of adverse childhood experiences. Child Maltreat.

[28] Shin SH, McDonald SE, Conley D (2018). Profiles of adverse childhood experiences and impulsivity. Child Abuse Negl.

