# Supplementary material for: Adverse childhood experiences and comorbidity in a cohort of people who have injected drugs
Source: BMC Public Health. 2022 May 16;22:986. doi: 10.1186/s12889-022-13369-5 (PMC9109307; doi:10.1186/s12889-022-13369-5)
Supplement: Supplementary file 1 — Additional file 1. [file 12889_2022_13369_MOESM1_ESM.docx]

**Additional file 1**

**Adverse Childhood Experiences and Comorbidity in a Cohort of People who Have Injected Drugs**

**Appendix**

**Appendix A**

**Procedure for Adverse Childhood Experiences Data Collection**

All ALIVE participants were eligible to participate in the present study if they attended an ALIVE study visit from August 1^st^ 2018 – December 31^st^ 2019 and had attended at least one prior ALIVE study visit. For this sub-cohort, 1,127 active ALIVE participants attended a study visit during this period and were eligible for this sub-study. Of these participants, 735 individuals were recruited and consented to participate in the sub-study. Thirteen participants were excluded because they subsequently declined to complete the section of the assessment examining child abuse (see below); an additional 69 participants were excluded due to missing data on any adverse childhood experience item, resulting in an analytic sample of 653 participants (see Appendix B).

All participants provided written informed consent to participate in this sub-study and were compensated with an additional $5. Six questions included in the adverse childhood experiences assessment asked participants about childhood experiences that likely constitute child abuse. Maryland State law requires that incidents of child abuse uncovered in the context of research and not previously reported to child protective services be reported by the researchers to the city Department of Social Services. For this reason, prior to asking participants’ these six questions, participants were told:

“In the next section, I’m going to ask some more questions about some things that an adult might have said or done to you before your 18^th^ birthday. Some of these things could indicate that, when you were a child, you experienced abuse. For this reason, if you answer yes to any of these next six questions, under Maryland State law, I will be obligated to make a report including your name and contact information to the Baltimore City Department of Social Services…Would you like me to proceed with this section?”

If participants chose to proceed, and endorsed any of the subsequent items, they were asked if that incident had previously been reported to a child protection agency. If no report had ever been made, a report was made to the Baltimore Department of Social Services, as required by Maryland law. Only 13 of 735 participants (< 2%) declined to complete this section after hearing this statement. The Johns Hopkins University institutional review board approved the data collection protocol for this sub-study, including the protocol covering breach of confidentiality associated with mandatory reporting.

Assessments were administered as part of an in-person interview by trained clinicians (a nurse and nurse-practitioner) and recorded electronically in a RedCap database during the interview. Participants’ childhood exposure to adversity and trauma was assessed using a modified version of the Adverse Childhood Experiences questionnaire.^1^ This assessment is based on the classic assessment administered by Felitti and colleagues to members of the Kaiser health system for the CDC’s Adverse Childhood Experience Study^2^ but adds four other common adverse experiences shown to predict poor outcomes.^1^ Fourteen adverse childhood experiences were assessed, with 21 questions: physical neglect (2 questions), emotional neglect (2 questions), physical abuse (2 questions), emotional abuse (2 questions), sexual abuse (2 questions), loss of a parent to divorce, abandonment or “some other reason” (1 question), growing up with domestic violence in the home (3 questions), having a parent with an alcohol or drug use problem (1 question), having a parent with mental illness (1 question), having a member of the household go to prison (1 question), being bullied by peers (1 question), being ostracized by peers (1 question), growing up in a violent neighborhood (1 question), and growing up in poverty (1 question). For adversities assessed with multiple items, endorsing any one of those items was sufficient to indicate the presence of that adversity. The survey items used to collect adverse childhood experiences data, and the associated scoring procedure, are presented in Appendices D and E, respectively.

**References**

1. Finkelhor D, Shattuck A, Turner H, Hamby S. A revised inventory of Adverse Childhood Experiences. *Child Abus Negl*. 2015;48:13-21. doi:10.1016/j.chiabu.2015.07.011

2. Felitti VJ, Anda RF, Nordenberg D, et al. Relationship of childhood abuse and household dysfunction to many of the leading causes of death in adults: The adverse childhood experiences (ACE) study. *Am J Prev Med*. 1998;14(4):245-258. doi:10.1016/S0749-3797(98)00017-8

**Appendix B**

**Sample Determination Flow Chart**

Eligible participants

(n = 1,127)

Eligible but not recruited (reason unknown)

(n = 392)

Recruited for the study

(n = 735)

Refused child abuse section (n = 13)

Missing data on any adversity item (n = 69)

Analytic sample

(n = 653)

**Appendix C**

**Comparison of Participants with and without Complete Adversity Data**

| Variable | Complete ACE Data  (n=653) | Missing ACE Data  (n = 69) | *p*-value |
| --- | --- | --- | --- |
| Race (% Black) | 81.1 | 82.6 | .87 |
| Sex (% male) | 67.3 | 78.2 | .07 |
| HIV (% positive) | 21.4 | 33.3 | .03 |
| HCV (% positive) | 76.7 | 71.0 | .29 |
| Current IDU (% yes) | 61.4 | 63.7 | .79 |
| Homelessness (% yes) | 25.1 | 34.7 | .08 |
| Income < $5,000/year (% yes) | 78.5 | 79.4 | 1.00 |
| Cohort 1 | 39 | 33 | .69 |
| Cohort 2 | 26 | 29 |  |
| Cohort 3 | 35 | 38 |  |
| Age (Median, IQR) | 47.5 (42.3-52.3) | 46.7 (39.9-53.2) | .46 |
| *Note*. IDU = injection drug use. IQR = interquartile range. Cohort 1 = recruitment period before 2005; Cohort 2 = recruitment period 2005-2014; Cohort 3 = recruitment period 2015-2018 | | | |

**Appendix D**

**Adverse Childhood Experiences Survey** **Items**

Response options:

No 0

Yes 1

Refused 7

Don’t Know 8

1. Before your 18^th^ birthday, did you often or very often feel that no one in your family loved you or thought you were important or special?

2. Before your 18^th^ birthday, did you often or very often feel that your family didn’t look out for each other, feel close to each other, or support each other?

3. Before your 18^th^ birthday, did you often or very often feel that you didn’t have enough to eat, had to wear dirty clothes, and had no one to protect you?

4. Before your 18^th^ birthday, did you often or very often feel that your parents were too drunk or high to take care of you or take you to the doctor if you needed it?

5. Before your 18^th^ birthday, was a biological parent ever lost to you through divorce, abandonment, or other reason?

6. Before your 18^th^ birthday, was your mother or stepmother often or very often pushed, grabbed, slapped, or had something thrown at her?

7. Before your 18^th^ birthday, was your mother or stepmother sometimes, often, or very often kicked, bitten, hit with a fist, or hit with something hard?

8. Before your 18^th^ birthday, was your mother or stepmother ever repeatedly hit over at least a few minutes or threatened with a gun or knife?

9. Before your 18^th^ birthday, did you live with anyone who was a problem drinker or alcoholic, or who used street drugs?

10. Before your 18^th^ birthday, was a household member depressed or mentally ill, or did a household member attempt suicide?

11. Before your 18^th^ birthday, did a household member go to prison?

12. Before your 18^th^ birthday, did other kids, including brothers or sisters, often or very often hit you, threaten you, pick on you or insult you?

13. Before your 18^th^ birthday, did you often or very often feel lonely, rejected or that nobody you?

14. Before your 18^th^ birthday, did you live for 2 or more years in a neighborhood that was dangerous, or where you saw people being assaulted?

15. Before your 18^th^ birthday, was there a period of 2 or more years when your family was very poor or on public assistance?

16. *Before your 18th birthday, did a parent or other adult in the household often or very often swear at you, insult you, put you down, or humiliate you?

17. *Before your 18th birthday, did a parent or other adult in the household often or very act in a way that made you afraid that you might be physically hurt?

18. *Before your 18^th^ birthday, did a parent or other adult in the household often or very often push, grab, slap, or throw something at you?

19. *Before your 18^th^ birthday, did a parent or other adult in the household ever hit you so hard that you had marks or were injured?

20. *Before your 18^th^ birthday, did an adult person at least 5 years older than you ever touch or fondle you or have you touch their body in a sexual way?

21. *Before your 18^th^ birthday, did an adult person at least 5 years older than you ever attempt or actually have oral, anal, or vaginal intercourse with you?

* If a participant responded “yes,” a follow-up question was asked, “To your knowledge, was this ever reported to any child welfare agency or law enforcement agency?”

**Appendix E**

**Scoring of Adverse Childhood Experiences Instrument**

| Adverse Childhood Experience | Questionnaire Item |
| --- | --- |
| 1. Emotional Neglect | 1 OR 2 |
| 1. Physical Neglect | 3 OR 4 |
| 1. Loss of Parent | 5 |
| 1. Domestic Violence in Home | 6, 7, OR 8 |
| 1. Parent Substance Use | 9 |
| 1. Parent Mental Illness | 10 |
| 1. Parent Incarcerated | 11 |
| 1. Bullying | 12 |
| 1. Social Ostracization | 13 |
| 1. Neighborhood Violence | 14 |
| 1. Poverty | 15 |
| 1. Emotional Abuse | 17 OR 18 |
| 1. Physical Abuse | 19 OR 20 |
| 1. Sexual Abuse | 21 OR 22 |
|  | |

**Appendix F**

**Most Prevalent Comorbid Conditions**

| Number of Conditions | Most Prevalent Condition(s) | Percent of Total Sample |
| --- | --- | --- |
| 0 Conditions | - | 33% |
| 1 Condition | Hypertension | 15% |
| 2 Conditions | Hypertension; Obesity | 6% |
| 3 Conditions | Hypertension; Obesity; Diabetes | 3% |
| 4 Conditions | Hypertension; Obesity; Diabetes; COPD | 1.1% |
| 5 Conditions | Hypertension; Obesity; Diabetes; COPD; Heart Disease | 0.4% |
| 6 Conditions | Hypertension; Obesity; Diabetes; COPD; Heart Disease; Stroke | 0.1% |
| 7 Conditions | Hypertension; Obesity; Diabetes; COPD; Heart Disease; Stroke; Renal Disease | < 0.1% |
| *Note*. COPD = Chronic obstructive pulmonary disease. | | |

**Appendix G**

**Results from Sensitivity Analysis**

| *Comorbidity Burden and Adverse Childhood Experiences in ALIVE among All Participants (N=722)* | | | | | | | |
| --- | --- | --- | --- | --- | --- | --- | --- |
| Outcomes | Exposures | Model A | | Model B* | | Model C** | |
| Comorbidity Groups | ACEs | OR (95% CI) | *p*-value | OR (95% CI) | *p*-value | OR (95% CI) | *p*-value |
| 0 comorbid conditions (ref.) | - | - | - | - | - | - | - |
| 1-2 comorbid conditions | 0 to 1 ACEs | ref. | - | ref. | - | ref. | - |
|  | 2 to 4 ACEs | 0.9 (0.6 – 1.2) | .40 | 0.9 (0.7 – 1.3) | .66 | 0.9 (0.7 – 1.3) | .72 |
|  | 5 to 9 ACEs | 0.9 (0.7 – 1.3) | .65 | 1.1 (0.8 – 1.6) | .63 | 1.1 (0.8 – 1.6) | .58 |
|  | ≥ 10 ACEs | 1.2 (0.7 – 2.0) | .46 | 1.2 (0.7 – 2.1) | .49 | 1.2 (0.7 – 2.1) | .44 |
| ≥ 3 comorbid conditions | 0 to 1 ACEs | ref. | - | ref. | - | ref. | - |
|  | 2 to 4 ACEs | 1.1 (0.7 – 1.8) | .74 | 1.3 (0.7 – 2.2) | .43 | 1.3 (0.7 – 2.3) | .38 |
|  | 5 to 9 ACEs | 1.2 (0.7 – 2.2) | .50 | 1.7 (0.9 – 3.2) | .09 | 1.8 (0.9 – 3.3) | .07 |
|  | ≥ 10 ACEs | 3.1 (1.5 – 6.4) | .002 | 3.0 (1.4 – 6.4) | .004 | 3.2 (1.5 – 6.9) | .003 |
| *Note*. ACEs = adverse childhood experiences. Model results reflect the inclusion of participants with missing values for adverse childhood experiences items, which were recoded to 0 = “No” (n = 722). OR = Odds Ratio. CI = Confidence Interval. *Estimates adjusted for age, race, sex, cohort, and past six-month income and homelessness. **Estimates additionally adjusted for HIV and anti-HCV status, and past six-month injection drug use. Estimates are based on the use of robust standard errors. | | | | | | | |
